# Pavement-DETR: A High-Precision Real-Time Detection Transformer for Pavement Defect Detection

**DOI:** 10.3390/s25082426

**Published:** 2025-04-11

**Authors:** Cuihua Zuo, Nengxin Huang, Cao Yuan, Yaqin Li

**Affiliations:** School of Mathematics and Computer Science, Wuhan Polytechnic University, Wuhan 430024, China; zuocuihua@whpu.edu.cn (C.Z.); hnx30@outlook.com (N.H.); yc@whpu.edu.cn (C.Y.)

**Keywords:** pavement defect detection, RT-DETR, accuracy improvement

## Abstract

The accurate detection of road defects is crucial for enhancing the safety and efficiency of road maintenance. This study focuses on six common types of pavement defects: transverse cracks, longitudinal cracks, alligator cracking, oblique cracks, potholes, and repair marks. In real-world scenarios, key challenges include effectively distinguishing between the foreground and background, as well as accurately identifying small-sized (e.g., fine cracks, dense alligator cracking, and clustered potholes) and overlapping defects (e.g., intersecting cracks or clustered damage areas where multiple defects appear close together). To address these issues, this paper proposes a Pavement-DETR model based on the Real-Time Detection Transformer (RT-DETR), aiming to optimize the overall accuracy of defect detection. To achieve this goal, three main improvements are proposed: (1) the introduction of the Channel-Spatial Shuffle (CSS) attention mechanism in the third (S3) and fourth (S4) stages of the ResNet backbone, which correspond to mid-level and high-level feature layers, enabling the model to focus more precisely on road defect features; (2) the adoption of the Conv3XC structure for feature fusion enhances the model’s ability to differentiate between the foreground and background, which is achieved through multi-level convolutions, channel expansion, and skip connections, which also contribute to improved gradient flow and training stability; (3) the proposal of a loss function combining Powerful-IoU v2 (PIoU v2) and Normalized Wasserstein Distance (NWD) weighted averaging, where PIoU v2 focuses on optimizing overlapping regions, and NWD targets small object optimization. The combined loss function enables comprehensive optimization of the bounding boxes, improving the model’s accuracy and convergence speed. Experimental results show that on the UAV-PDD2023 dataset, Pavement-DETR improves the mean average precision (mAP) by 7.7% at IoU = 0.5, increases mAP by 8.9% at IoU = 0.5–0.95, and improves F1 Score by 7%. These results demonstrate that Pavement-DETR exhibits better performance in road defect detection, making it highly significant for road maintenance work.

## 1. Introduction

With the continuous progress of urbanization, roads, as an essential component of transportation infrastructure, bear the crucial responsibility of daily transportation and residents’ travel. However, due to long-term traffic load, climate change, and natural aging, road surfaces often develop cracks, potholes, and other types of damage. These defects not only severely impact traffic safety but also significantly reduce the travel experience for residents. Therefore, establishing an efficient Pavement Management System (PMS) [1] is of great significance for ensuring traffic safety, extending the lifespan of roads, and improving road maintenance efficiency.

Traditional road defect detection primarily relies on manual inspections. Although this method can identify many obvious defects, it faces significant limitations in terms of efficiency and worker safety, especially in complex road environments, harsh weather conditions, and large-scale data processing. Manual inspections not only consume a large amount of human and material resources but are also constrained by the experience and subjective factors of the inspectors, making it difficult to ensure the detection’s efficiency and accuracy, and preventing a comprehensive and objective assessment of the extent of road damage. Furthermore, as road networks continue to expand, traditional manual inspection methods are no longer sufficient to meet the demands of large-scale pavement management.

In recent years, computer vision technology, particularly in the field of object detection, has developed rapidly. The introduction of numerous innovative models has significantly driven the advancement of this field, offering fresh insights into deep learning-based automated pavement defect detection. Based on the characteristics of the algorithmic workflow, deep learning-based object detection models can be mainly divided into two categories: one-stage object detection algorithms and two-stage object detection algorithms.

Two-stage object detection algorithms first generate candidate regions that are likely to contain objects, and then perform more refined classification and regression on these regions. Representative models of this approach include the R-CNN series (Region-based Convolutional Neural Networks), such as R-CNN [2], Fast R-CNN [3], Faster R-CNN [4], Mask R-CNN [5], and Cascade R-CNN [6]. Although two-stage algorithms perform relatively well in terms of accuracy, particularly with small object detection, their real-time detection performance is limited due to long computation times and complex model structures, making them less suitable for applications that require high efficiency.

To address the time consumption issue of two-stage algorithms, one-stage object detection algorithms, represented by the SSD [7] and YOLO (you only look once) [8,9,10,11,12] series, have emerged. These algorithms directly predict the class and location of the object, eliminating the need for generating candidate regions, which significantly speeds up the training process and meets the real-time detection requirements. With continuous innovations in feature extraction, bounding box prediction, and other areas, the accuracy and speed of one-stage algorithms have been consistently optimized and improved.

Meanwhile, the Transformer architecture, initially applied in the field of natural language processing (NLP), demonstrated outstanding capabilities in modeling long-range dependencies and parallel computation, attracting significant attention from the academic community. Subsequently, Carion et al. [13] introduced it to object detection tasks and proposed the DETR model. By fully utilizing Transformer’s global modeling capabilities, DETR eliminated the reliance on anchor boxes and non-maximum suppression used in traditional object detection methods, achieving a true end-to-end object detection model. This innovation greatly simplified the training and deployment processes, opening up a new research direction in the field of object detection. Following this, scholars proposed numerous improvements in training efficiency, detection accuracy, and handling complex scenes, leading to the development of variants such as Deformable-DETR [14], Dab-DETR [15], Conditional-DETR [16], and Dino-DETR [17].

These variants significantly improved the model’s detection accuracy while reducing its complexity to some extent. However, due to the inherently high computational complexity of the Transformer architecture, DETR and its variants have always struggled to meet real-time requirements, especially when compared to the YOLO series models, where the trade-off between detection speed and accuracy is more pronounced. To address this issue, Zhao et al. [18] proposed the RT-DETR model, which innovatively incorporates efficient hybrid encoders, IoU-aware query selection, and adaptive inference speed techniques, significantly enhancing both detection accuracy and speed, and successfully achieving true real-time performance.

**Figure 1:**
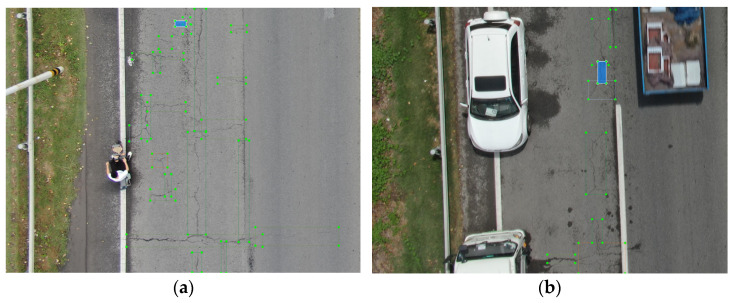
Examples of challenges in pavement defect detection: (**a**) illustration of small defect targets distributed with substantial overlap; (**b**) illustration of background interference.

However, RT-DETR still has some limitations when applied to road defect detection. Firstly, road surface defect detection often relies on image acquisition from drones or other sensing platforms such as vehicle-mounted or roadside cameras. In this study, we focus on UAV-based imagery, which commonly contains small targets and overlapping objects due to the high-altitude perspective. As shown in Figure 1a, the image illustrates many small defect targets intertwined on the road. RT-DETR performs suboptimally in detecting small and overlapping targets, especially in complex backgrounds. Secondly, during drone image capture, factors such as vehicles, pedestrians, and pets can cause interference. As shown in Figure 1b, the image demonstrates the potential interference caused by objects such as vehicles during the capturing process, which may affect object detection. In environments with significant background interference, RT-DETR struggles with distinguishing the foreground from background, which leads to a decrease in detection accuracy.

To address the aforementioned issues, we have designed and improved an RT-DETR-based road defect detection model called Pavement-DETR, selecting ResNet18 [19] as the backbone network to reduce the model’s complexity. The design of this model is optimized in the following three aspects:

(1) We introduce a CSS attention mechanism composed of channel attention, channel shuffle, and spatial attention at the S3 and S4 feature layers. This mechanism reduces redundant information between channels, enhances information fusion across different channels, and simultaneously captures spatial dependencies, enabling the model to focus more on learning road defect features.
(2) We improve the feature fusion module by adopting a Conv3XC structure, combining multi-level convolutions, channel expansion, and skip connections. This improvement significantly enhances the model’s feature extraction capabilities, especially in complex scenarios, enabling better differentiation between road defects and other objects. Additionally, this design strengthens gradient flow, ensuring stable training and efficient inference, thus meeting real-time detection requirements.
(3) We propose a loss function that combines PIoU v2 and NWD weighted averaging. By combining these two, the loss function simultaneously optimizes the overlapping part, and the position and size of the bounding boxes, thereby improving the model’s accuracy comprehensively. Moreover, this weighted loss function smooths the optimization process, promotes a faster convergence of the model, and improves training efficiency and final detection performance.

Through the above design improvements, we aim to significantly enhance overall accuracy while maintaining real-time performance. Finally, we validate the effectiveness of the Pavement-DETR model through a series of experiments, fully demonstrating its potential in road defect detection tasks.

The structure of this paper is as follows: Section 2 provides an overview of the research status in road defect detection both domestically and internationally, and reviews the related research progress of RT-DETR; Section 3 details the design and improvements of the Pavement-DETR model; Section 4 presents various experiments and their results analysis; and Section 5 summarizes the main conclusions of this paper and outlines future research directions.

## 2. Related Work

### 2.1. Pavement Defect Detection

Pavement defect detection can be mainly divided into two categories: traditional pavement defect detection methods and deep learning-based pavement defect detection methods. Traditional detection methods include threshold segmentation, edge detection, region growing methods, and traditional machine learning methods such as neural networks and support vector machines for crack detection. These traditional algorithms played an important role in the early stages but typically rely on manually designed features and demonstrate poor robustness in complex environments. For instance, they struggle with challenges such as variations in lighting and different road surface materials. Moreover, these methods are susceptible to environmental noise interference, such as traffic and weather conditions, as well as signal noise in image processing. This leads to unstable detection results, often resulting in missed or false detections [20].

With the rapid development of deep learning, new approaches have been introduced to the field of pavement defect detection, becoming the key to addressing these challenges. Deep learning-based pavement defect detection methods can be primarily categorized into two types: methods based on 2D images and methods based on 3D images. Deep learning-based 3D image methods typically train deep neural networks using high-precision pavement point cloud data, greatly enhancing the accuracy and efficiency of defect detection. However, these methods often require large amounts of labeled data and powerful computational resources, making them costly and challenging for practical applications. In contrast, 2D image-based deep learning methods offer greater practicality. Two-dimensional images significantly reduce computational overhead, while deep neural network-based learning methods still maintain high accuracy, striking a balance between cost and efficiency. Against this backdrop, many researchers have proposed deep learning models based on 2D images to improve the effectiveness and efficiency of pavement defect detection. For example, Maeda et al. [21] proposed a pavement defect detection model based on the SSD network, which achieved both detection and localization of pavement defects. At the same time, a weakly supervised labeling method was used to generate a dataset covering various weather and lighting conditions. Nevertheless, the model’s localization accuracy is limited by its relatively simple feature fusion approach, making it difficult to handle more complex detection tasks. Gou et al. [22] proposed a pavement defect detection method based on an improved Faster R-CNN. This method combines an improved feature extraction network and a regional proposal network, effectively overcoming issues such as low efficiency, poor generalization under lighting variations, and interference from image noise, which are common in traditional pavement defect detection methods. Training and testing on the collected crack image dataset HTD showed that this method can achieve high detection accuracy. Yet, the model still faces issues with high computational overheads and slow processing speeds. Shen et al. [23] proposed a pavement defect detection method based on an improved Cascade R-CNN. This method is capable of classifying five types of pavement defects and effectively addresses the shortcomings of traditional pavement damage detection systems in terms of insufficient classification and low accuracy. Training and testing on the processed dataset demonstrated its superior detection performance. With the clear performance advantages of single-stage object detection algorithms, their simplified structure and higher computational efficiency have attracted significant attention from researchers. As a result, an increasing number of studies are focusing on the exploration and application of these algorithms. Xiang et al. [24] proposed an improved YOLOv5-CBoT network that combines the Bottleneck Transformer and C2f module for pavement defect detection. This method effectively captures long-range dependencies and adapts to detection tasks involving cracks and other long-span, slender features. Experimental results on the RDD2020 dataset showed that the improved network not only significantly reduced the number of parameters but also enhanced detection speed and accuracy, demonstrating superior performance. With the continuous advancement of the YOLO model series, researchers have consistently optimized and improved its structure to meet the requirements of high accuracy, lightweight design, and real-time performance in the field of pavement defect detection. Wang et al. [25] proposed a pavement defect detection algorithm based on an improved YOLOv8s, named BL-YOLOv8. This method reconstructs the original model’s Neck structure by integrating the BiFPN method, optimizing feature fusion capabilities; introduces the SimSPPF module to improve detection speed; and employs a dynamic large convolution kernel attention mechanism, LSK-attention, to expand the model’s receptive field and enhance detection accuracy. Through these improvements, BL-YOLOv8 not only boosts detection performance but also reduces the number of parameters and computational load, significantly enhancing the model’s efficiency and adaptability. Youwai et al. [26] proposed a lightweight pavement defect detection model named YOLO9tr. Based on YOLOv9, YOLO9tr introduces a partial attention block, effectively improving detection accuracy and inference speed in complex scenarios, making it suitable for real-time pavement monitoring applications. To address the demand for real-time detection in resource-constrained scenarios, Ou et al. [27] proposed GASYOLO, a lightweight pavement defect detection model based on an improved YOLOv10 framework. By incorporating optimization strategies such as feature generation, asymmetric convolution, and grouped channel shuffle, the model effectively enhances multi-scale feature representation. GASYOLO significantly reduces parameters and computational complexity while maintaining high detection accuracy, making it more suitable for practical deployment.

### 2.2. Transformer-Based Object Detection Network

Transformer was initially used for natural language processing until Vision Transformer introduced it to the field of computer vision, breaking the traditional reliance on convolutional neural networks in this domain. Vision Transformer achieved state-of-the-art performance in several image classification tasks by dividing images into patches and applying self-attention mechanisms.

Based on the success of Vision Transformer, Dosovitskiy et al. [28] proposed DETR, which was the first model to apply Transformer to the field of object detection. Leveraging the global modeling capability of Transformer, DETR can directly generate bounding boxes and classification results, achieving end-to-end object detection. This model demonstrates performance comparable to traditional convolutional neural network-based models.

However, DETR suffers from slow training convergence and poor performance in detecting small objects (defined as those where the square root of the ratio of the bounding box area to the image area is less than 0.03). To address these issues, Carion et al. [13] proposed Deformable DETR, which introduces innovative techniques such as Deformable Convolutions, Deformable Attention, and multi-scale feature fusion. The Deformable Attention mechanism focuses on key features in specific regions rather than performing global computation, significantly reducing the computational load and accelerating the training process. Additionally, multi-scale features and encoding at different levels effectively improve the detection accuracy of small objects.

Nevertheless, the learnable object queries in the above DETR variants only provide reference information for the center point of the object bounding box without considering the actual width and height of the object. To address this issue, Liu et al. [15] proposed DAB-DETR, which reintroduces anchor boxes into the DETR framework. This allows the model to better cover objects of different sizes, enhances the interpretability of the queries, and accelerates the convergence speed during training.

Building on the advantages of the aforementioned DETR variants, Zhang et al. [17] proposed the DINO model, which introduces innovative techniques such as Contrastive Denoising Training, Mixed Query Selection, and Look Forward Twice. These innovations significantly improve both training efficiency and detection performance. Contrastive Denoising Training reduces duplicate predictions and enhances the detection of small objects; Mixed Query Selection optimizes query selection, boosting accuracy; and Look Forward Twice accelerates training convergence. Overall, these techniques enable DINO to retain a simplified training process while overcoming the drawbacks of long training times and poor small object detection.

The aforementioned DETR variants have significantly improved detection accuracy and reduced model complexity, but due to the high computational complexity of the Transformer structure, the issue of high computational cost remains unresolved, making it unsuitable for real-time detection tasks.

To address this challenge, Zhao et al. [18] proposed RT-DETR, which introduces several innovative techniques, including an efficient hybrid encoder, IoU-aware query selection, and adaptive inference speed. The efficient hybrid encoder optimizes multi-scale feature processing by decoupling within-scale interactions and cross-scale feature fusion, reducing computational costs and supporting real-time object detection. IoU-aware query selection improves the initialization of target queries, allowing the model to focus on the most relevant objects, thereby enhancing detection accuracy. An adaptive inference speed adjusts the inference speed flexibly by using different decoder layers without the need for retraining. These combined innovations enable RT-DETR to achieve significant advantages in both accuracy and speed for real-time object detection. Therefore, RT-DETR is chosen as the baseline model in this study.

## 3. Method

### 3.1. RT-DETR

In April 2023, Baidu’s research team introduced the revolutionary object detection model, RT-DETR. This model marks the first implementation of end-to-end real-time object detection, preserving the post-processing-free benefits of the DETR series while also fulfilling the stringent real-time requirements demanded by industrial applications.

The development of RT-DETR centers around three core innovations. Firstly, it utilizes an Efficient Hybrid Encoder design capable of adeptly handling multi-scale features. This design includes the decoupling of intra-scale feature interactions and the integration of a cross-scale feature fusion module. These mechanisms not only significantly reduce the computational load but also enhance the model’s capability to accurately process targets of varying sizes. Secondly, the model introduces an IoU-Aware Query Selection mechanism that provides high-quality initial object queries to the decoder, thereby optimizing the precision and response speed of the object detection. Lastly, RT-DETR incorporates Adaptive Inference Speed technology, which allows for flexible adjustments of the inference speed without retraining, providing robust support for various real-time application scenarios and ensuring optimal performance in different environments.

**Figure 2:**
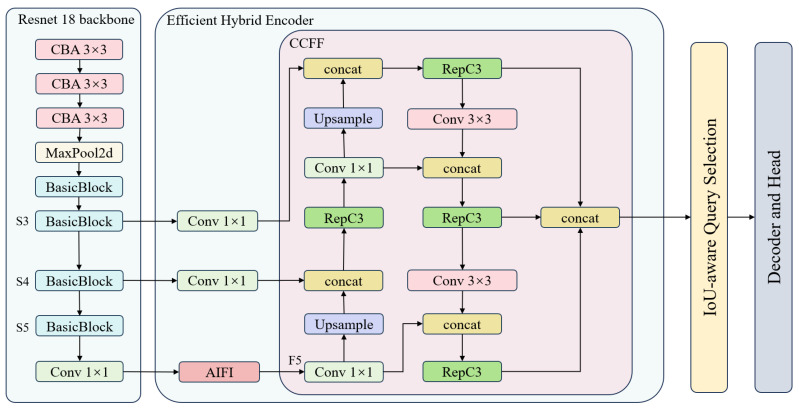
Network architecture of RT-DETR. S3, S4, and S5 represent the third, fourth, and fifth feature levels of the backbone network, respectively. F5 is an additional high-level feature layer. These layers are enhanced for multi-scale object detection by using Attention-based Intra-scale Feature Interaction (AIFI) and CNN-based Cross-scale Feature Fusion (CCFF).

RT-DETR offers several model configurations to suit different application scenarios. Among these, RT-DETR-L, RT-DETR-X, and RT-DETR-R50 have shown outstanding performance on the COCO val2017 dataset. This study selects RT-DETR-R18 as the baseline model for enhancement. The architecture of this model is clearly divided into three parts: the backbone, encoder, and decoder, as illustrated in Figure 2.

### 3.2. Pavement-DETR

Although RT-DETR exhibits outstanding performance in general object detection tasks, it encounters specific challenges in the context of road defect detection. The model particularly struggles with detecting small or overlapping objects and distinguishing between the foreground and background in environments with complex backdrops.

**Figure 3:**
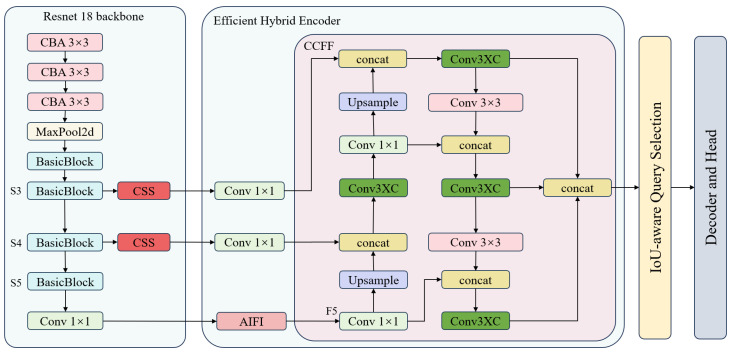
Pavement-DETR Network framework.

To address these challenges, this paper introduces the Pavement-DETR model, a refined model specifically designed to enhance accuracy in road defect detection. Initially, we incorporated the CSS attention mechanism which, by boosting the model’s capability to express features, allows it to focus more acutely on crucial road defect characteristics, significantly enhancing detection accuracy. Secondly, to more effectively handle background interference, we implemented the Conv3XC feature fusion module, which strengthens the exchange of information between different feature layers, greatly improving the model’s ability to distinguish between the foreground and background. Lastly, in response to the challenges of detecting small and overlapping objects, we developed a new loss function that combines the features of PIoU v2 and NWD. This loss function is specially designed to optimize the handling of overlapping bounding boxes and the adjustment of their positions and sizes. By imposing stricter penalties on the detection and positioning of small objects, it substantially increases the precision in identifying small objects and also enhances the model’s capability to recognize overlapping objects. The improved model structure is illustrated in Figure 3.

#### 3.2.1. CSS Attention Mechanism

In the early stages of the development of Pavement-DETR, we observed that while the original RT-DETR model could identify the approximate location of road defects, its feature maps exhibited low discriminability between the target and background. The features of the target region and the background were mixed, making it difficult for the model to accurately extract the key characteristics of road defects. This limitation not only affected the model’s detection accuracy but also increased the risk of missed and false detections.

**Figure 4:**
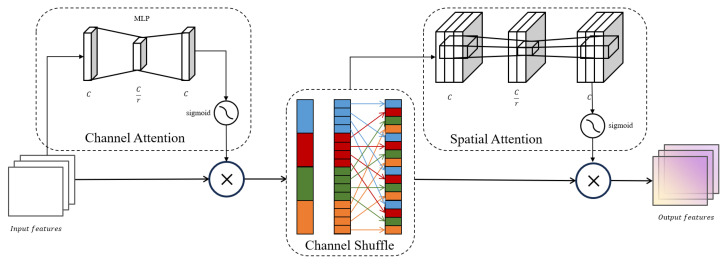
CSS Attention Mechanism framework.

To address this issue and enhance the model’s ability to capture features and improve recognition accuracy, we propose a CSS attention mechanism composed of channel attention, channel shuffle [29], and spatial attention mechanisms. These components work together to make the model more attentive to road defect features and reduce its response to surrounding noise. The structural diagram of the CSS attention mechanism is shown in Figure 4.

1. Channel attention: This module utilizes a two-layer multilayer perceptron to capture the interdependencies among feature map channels. Initially, the first layer of the MLP reduces the number of channels using a preset compression ratio, aimed at decreasing the model’s complexity and mitigating the risk of overfitting. Subsequently, a ReLU activation function introduces necessary non-linearity, enhancing the model’s capability to process features. The second layer of the MLP then restores the number of channels to the original size to preserve essential feature information. After this process, the output is mapped to a range between 0 and 1 through a Sigmoid activation function, creating a finely tuned channel attention map. This attention map is then element-wise multiplied with the original feature map, effectively adjusting the contribution of each channel and emphasizing features in critical channels, thereby significantly improving the model’s ability to recognize features in complex scenarios.
2. Channel shuffle: This module is a key component of the CSS attention mechanism. Its main function is to enhance the exchange and sharing of information between different channels by rearranging the order of channels in the input feature map. In operation, the channels of the feature map are first grouped according to a preset number of groups. Then, a transpose operation is performed, which rearranges the order of channels within each group, breaking the original organizational structure and facilitating better integration of cross-channel features. This reorganized channel structure allows the model to gather information from a broader context when processing subsequent features, enhancing its ability to capture complex features.
3. Spatial attention: This module utilizes two 7 × 7 large convolutional kernels to enhance the model’s ability to capture spatial details of feature maps and improve its contextual understanding. Initially, the first 7 × 7 convolutional kernel processes the input feature map, reducing the number of channels to a predetermined value. This not only significantly reduces the model’s parameter count but also, with the broad spatial coverage of the large kernel, helps capture more extensive contextual information. Subsequently, batch normalization is employed to stabilize the training process, and a ReLU activation function is introduced to increase non-linearity, enabling the model to better recognize complex spatial patterns. Then, the second 7 × 7 convolutional kernel restores the channel count to its original scale, ensuring the integrity of feature information. Finally, a spatial attention map generated by the Sigmoid function is element-wise multiplied with the original feature map, allowing the model to adjust its focus at each position of the feature map based on the weights of spatial attention, thus more accurately highlighting critical areas and enhancing overall recognition accuracy.

In the design of the CSS attention mechanism, the unique and complementary functions of each submodule collectively optimize the overall performance of the model. The channel attention mechanism first enhances the model’s global information capture by precisely adjusting the weights of each channel. Subsequently, the channel shuffle mechanism ensures effective interaction among the enhanced features across channels, further enriching feature expression. Finally, the spatial attention mechanism compensates for the shortcomings of relying solely on channel information by focusing on the spatial details of images, thus enhancing the model’s sensitivity to local features. The combination of these three mechanisms not only improves the efficiency and accuracy of the model in capturing key features but also significantly enhances its recognition capabilities through a deep integration of channel and spatial dimensions.

In the Pavement-DETR model, we decided to introduce the CSS attention mechanism into the S3 and S4 feature layers to significantly enhance their feature extraction capabilities, thereby improving the overall detection accuracy of the model.

#### 3.2.2. Conv3XC

**Figure 5:**
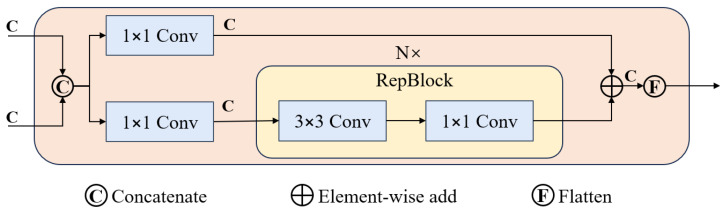
RT-DETR Feature Fusion Module, where C represents the number of channels in the input feature map.

In the RT-DETR model, feature fusion plays a crucial role. The model’s design includes an Efficient Hybrid Encoder that repeatedly utilizes feature fusion technology to integrate features from various scales, significantly enhancing the model’s ability to capture details in complex image scenes. In this process, the RepBlock module is the core part of feature fusion. This module consists of a 3 × 3 convolution and a 1 × 1 convolution, and Figure 5 depicts the complete architecture of feature fusion in RT-DETR. The 3 × 3 convolution primarily captures spatial information from the input features, effectively extracting key details from the images; meanwhile, the 1 × 1 convolution is mainly used to adjust the number of channels, aiming to reduce the model’s parameter count and computational complexity, thus optimizing overall network efficiency. During the deployment phase, RepBlock employs an efficient fusion strategy, merging these two convolutional layers into one, significantly enhancing inference speed.

**Figure 6:**
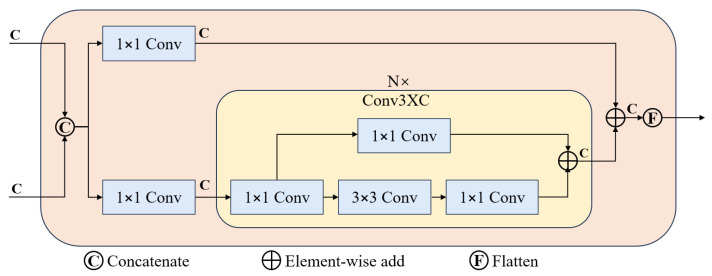
Pavement-DETR Feature Fusion Module, where C represents the number of channels in the input feature map.

The Conv3XC [30] module consists of a three-tier convolutional structure and a skip connection, with Figure 6 depicting the complete architecture of feature fusion in Pavement-DETR. In this module, feature maps first undergo three stages of convolution to expand and compress the number of channels. This design not only effectively captures the underlying detail information from the input features but also integrates higher-level abstract information. Additionally, the skip connection in the module directly links the input to the output, closely integrating with the convolutional path, significantly enhancing the integrity and efficiency of information transmission. This residual structure effectively reduces information loss during feature transformation, enhancing the model’s ability to fuse and recognize features against complex backgrounds. During model deployment, Conv3XC employs an efficient fusion technique that combines the weights of the convolutional layers and the skip connection to optimize inference efficiency.

#### 3.2.3. Improved Loss Function

Loss functions play a crucial role in the training process of models, defining the inconsistency between model outputs and actual labels and guiding the model on how to optimize its parameters to reduce this inconsistency. In the RT-DETR model, the GIoU loss function is adopted. It is simple and efficient, capable of simultaneously focusing on overlapping and non-overlapping areas, thus more precisely measuring the overlap between the predicted and actual bounding boxes. Moreover, by introducing the concept of the minimum enclosing box, GIoU effectively addresses the issue of gradient disappearance when the predicted and actual bounding boxes do not overlap at all, significantly enhancing the model’s performance in bounding box localization.

Although the GIoU loss function performs well in handling non-overlapping bounding boxes, its effectiveness degrades to that of traditional IoU when the prediction box is entirely within the true box, at which point it fails to effectively distinguish the relative position between the predicted and actual boxes. Moreover, GIoU’s performance in optimizing small and overlapping targets is mediocre. To address these limitations, we propose an improved loss function that combines PIoU v2 [31] and NWD [32], with the overall calculation method of this combined loss function shown in Equation (1).

(1) $$L_{\mathrm{combined}}=iou\_ratio\cdot \left(\frac{\sum L_{{PIoU}_{v2}}}{N}\right)+\left(1-iou\_ratio\right)\cdot \left(\frac{\sum \left(1.0-NWD\right)}{N}\right)$$

In Equation (1), iou_ratio determines the relative contribution ratio of PIoU v2 loss to NWD loss in the total loss function. Based on experimental experience and parameter sensitivity analysis, the iou_ratio was set to 0.45 in our experiments to achieve optimal detection performance. $\sum L_{PIoU_{v}2}$ denotes the sum of the PIoU v2 loss functions for all samples, N represents the number of bounding boxes, and $\sum \left(1.0-NWD\right)$ represents the sum of 1 minus the normalized Wasserstein distance for all samples. By combining the PIoU v2 and NWD loss functions through a weighted average, the model can be optimized simultaneously in terms of localization accuracy and bounding box shape alignment, effectively enhancing the model’s precision in complex environments.

Specifically, the PIoU v2 loss function is an extension of the PIoU loss function, integrating the PIoU loss function’s target size-adaptive penalty factor and a gradient-adjusting function based on anchor box quality, while also introducing an innovative non-monotonic attention layer. This target size-adaptive penalty factor and gradient-adjusting function based on anchor box quality work together to greatly simplify the parameter adjustments during the model regression process, thereby accelerating the convergence of the model. Additionally, the non-monotonic attention layer enhances the model’s capability to focus on anchor boxes of medium quality, further improving detection accuracy. Overall, the PIoU v2 loss function not only optimizes the efficiency of model training but also significantly enhances the overall accuracy of the model in complex scenarios.

The NWD loss function introduces a novel metric based on the Normalized Wasserstein Distance. Specifically, it models both predicted and ground-truth bounding boxes as two-dimensional Gaussian distributions, with the mean representing the bounding box center and the covariance matrix determined by the width and height of the bounding box. The similarity between the predicted and ground-truth boxes is calculated using the Wasserstein distance between their respective Gaussian distributions, and this value is further normalized, resulting in a stable and scale-invariant measure of bounding box similarity. This method is particularly effective in addressing common challenges in small object detection, such as when the predicted and actual boxes do not overlap or only partially overlap. Compared to the traditional IoU series, NWD offers more stable scale invariance performance, making it more suitable for assessing the loss of small-scale targets. Overall, the NWD loss function significantly enhances the accuracy of models in detecting small objects.

To maximize the advantages of both the PIoU v2 and NWD loss functions, we have proposed a fusion strategy that combines them through a weighted average approach. This innovative combination not only significantly enhances the model’s accuracy in detecting small objects but also strengthens its capability in handling overlapping targets and making precise adjustments. In this way, we effectively overcome the limitations associated with relying on a single loss function, providing a more comprehensive and efficient solution for model optimization.

## 4. Experiment and Result

### 4.1. Dataset

This study uses the UAV-PDD2023 dataset [33], which is open-sourced by Hebei University of Technology. The dataset consists of 2440 high-resolution, three-channel JPG images (2592 × 1944 pixels) captured by drones on various roads across China, covering a total of 10,006 instance labels. The dataset includes images taken under two weather conditions: clear skies and one hour after rain, as well as three types of road categories: highways, provincial roads, and county roads. This diversity effectively showcases road conditions under different real-life scenarios, enhancing the dataset’s practicality.

In addition, the dataset is divided into six typical road defect categories: longitudinal cracks, transverse cracks, alligator cracks, oblique cracks, repair, and potholes. To enable effective training and evaluation of the model, the dataset is split into three subsets: 1561 images for training, 391 images for validation, and 488 images for testing.

### 4.2. Experimental Environment

All model improvements and experiments were conducted under the same hardware and environment, with the specific device configuration shown in Table 1.

All model improvements and experiments were conducted using the same hyperparameter settings. The detailed hyperparameter configuration is shown in Table 2.

### 4.3. Evaluation Indicators

To accurately evaluate the improvements of the Pavement-DETR model, this study uses several key performance metrics, including Precision (P), Recall (R), F1 Score, Average Precision (AP), mean Average Precision (mAP), and giga floating-point operations per second (Gflops), to ensure a comprehensive evaluation of the model’s performance. The specific details of these metrics are as follows.

The confusion matrix provides an intuitive method to illustrate the relationship between the model predictions and actual outcomes. In this matrix, ‘True’ and ‘False’ indicate the model’s predictions, while ‘Positive’ and ‘Negative’ represent the actual conditions. Following this classification, the confusion matrix is divided into four parts: True Positives (TP), False Positives (FP), False Negatives (FN), and True Negatives (TN).

Precision represents the proportion of actual positive instances among all the predicted boxes that the model classifies as positive. Higher Precision means the model is more accurate in predicting positive instances, with fewer false positives. Its formula is as follows:

(2) $$P=\frac{\mathrm{TP}}{\mathrm{TP}+\mathrm{FP}}$$

Recall represents the proportion of actual positive instances that are correctly identified by the model. Higher Recall means the model can capture most of the true positive instances, with fewer false negatives. Its formula is as follows:

(3) $$R=\frac{\mathrm{TP}}{\mathrm{TP}+\mathrm{FN}}$$

F1 Score is the harmonic mean of Precision and Recall. It considers both the accuracy and the coverage of the model in predicting the positive class, making it suitable for imbalanced class situations. Its formula is as follows:

(4) $$F1\;\mathrm{Score}=2\times \frac{P\times R}{P+R}$$

AP is the area under the P-R curve, reflecting the average accuracy of a single-class model at different recall rates. Its formula is as follows:

(5) $$\mathrm{AP}=\int_{0}^{1}P\left(R\right)\;dR$$

mAP is the average of the APs for each category, providing a comprehensive evaluation of the model’s performance across all categories. The calculation formula is as follows. In this study, mAP@0.5 and mAP@0.5:0.95 are used as evaluation metrics, where mAP@0.5 refers to the mAP calculated with an IoU threshold of 0.5, and mAP@0.5:0.95 is the average AP computed at multiple IoU thresholds ranging from 0.5 to 0.95.

(6) $$\mathrm{mAP}=\frac{1}{N}\sum_{i=1}^{N}AP_{i}$$

### 4.4. Analysis of Experimental Results

#### 4.4.1. Performance Evaluation

In this study, we selected several SOTA object detection algorithms for comparison experiments to comprehensively evaluate the performance of our Pavement-DETR model, which is based on the RT-DETR-R18 architecture. The results of the comparison experiments are shown in Table 3. The compared models include the popular YOLO series (YOLOv8s, YOLOv8m, YOLOv9s, YOLOv9m, YOLOv10s, and YOLOv10m) as well as the increasingly popular DETR variants (Deformable-DETR, Dab-DETR, Conditional-DETR, and Dino-DETR). Through these comparison experiments, we found that compared to the baseline model RT-DETR-R18, Pavement-DETR achieved a 7.7% improvement in mAP50 and an 8.9% improvement in mAP50–95 with an increase of 10.5 Gflops in computational cost, demonstrating a significant accuracy improvement without a substantial increase in computational load. Compared to other SOTA object detection models, although YOLOv10s showed the lowest computational cost, Pavement-DETR outperformed all the compared models in terms of Precision, Recall, mAP50, and mAP50–95. This result further validates the effectiveness of our improvements and highlights the significant gains in accuracy and performance achieved by the modified Pavement-DETR model. The experimental results show that Pavement-DETR offers higher detection accuracy for pavement defects and effectively balances the trade-off between computational cost and precision, demonstrating excellent performance and strong practical applicability.

#### 4.4.2. Ablation Experiment

To systematically validate the effectiveness of each improvement we proposed, we designed ablation experiments. The experimental setup involved progressively introducing each improvement while keeping the configuration constant, and analyzing the specific contribution of each measure to the model’s performance. The results of the ablation experiments on the test set are shown in Table 4.

As shown in Table 4, compared to the baseline model, the introduction of the CSS attention mechanism improved the model’s mAP50 by 1.9%, mAP50–95 by 3.9%, and F1 Score by 1%. Despite an increase of 10.5 Gflops in computational cost, it effectively enhanced the model’s feature extraction capability, leading to more precise localization. The addition of the Conv3XC module significantly improved model performance without increasing computational cost, with mAP50 increasing by 3.9%, mAP50–95 by 3.6%, and F1 Score by 5%. This module effectively improved the model’s feature fusion ability, helping the model better distinguish between the foreground and background of the image. Furthermore, the improved loss function also further boosted the model’s performance without adding computational cost, with mAP50 increasing by 1.9%, mAP50–95 by 1.4%, and F1 Score by 1%. This improvement enhanced the model’s ability to detect small objects and strengthened its focus on medium-to-high-quality anchor boxes, thereby improving training efficiency. The CSS attention mechanism effectively enhances the model’s feature representation capability, providing a solid foundation for cross-scale feature fusion by the Conv3XC module. Subsequently, the Conv3XC module efficiently integrates multi-scale features, resulting in more comprehensive and precise feature representations, thereby further strengthening the performance of the CSS attention mechanism. Moreover, the high-quality features produced by these modules improve the optimization efficiency of the loss function during the regression stage, significantly enhancing the training effectiveness of the model. Consequently, the integrated combination of these three modules achieves a synergistic enhancement of overall model performance, substantially improving detection accuracy without significantly increasing computational complexity. Based on the ablation experiment results, we can clearly see the specific contribution of each improvement to the model’s performance, further validating the effectiveness of these improvements in enhancing both accuracy and efficiency.

#### 4.4.3. Grad-CAM++ Visualization

To more intuitively demonstrate the detection performance of the improved model, we employ the Grad-CAM++ method for model visualization analysis. Grad-CAM++ generates heatmaps to highlight the regions the model focuses on when making decisions, thereby providing a visual interpretation of the decision-making process.

**Figure 7:**
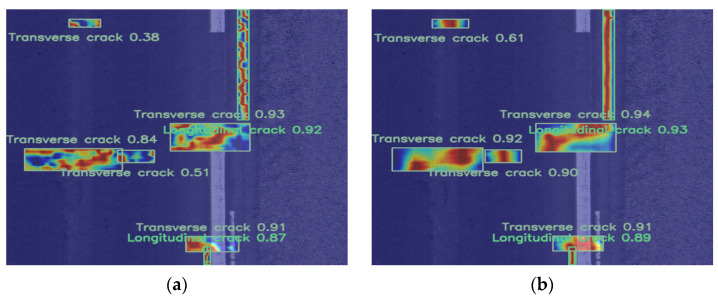
Heatmap comparison example: (**a**) RT-DETR Heatmap; (**b**) Pavement-DETR Heatmap.

As shown in Figure 7a, the heatmap generated by the RT-DETR model exhibits noticeable issues. Specifically, the color distribution in the heatmap is overly diffuse, resulting in abnormally high temperature values in areas adjacent to actual cracks. This indicates that the RT-DETR model has limitations in spatial resolution, failing to accurately capture the specific features of road defects. Consequently, it struggles to effectively distinguish defect regions from non-defect regions, increasing the risk of missed detections and false positives.

In contrast, as shown in Figure 7b, the heatmap generated by Pavement-DETR exhibits clearer and more distinct road defect features. Compared to the heatmap generated by RT-DETR, the Pavement-DETR heatmap not only captures more detailed information and focuses more precisely on defect areas but also reduces false detections. This result demonstrates that Pavement-DETR has significant advantages in detection performance and can more effectively identify road defects.

#### 4.4.4. Robustness Evaluation

**Figure 8:**
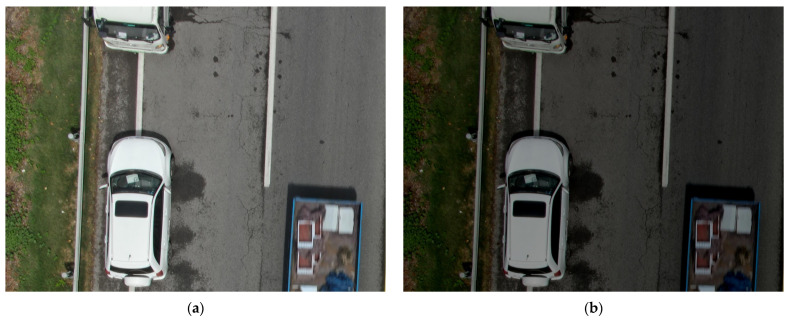

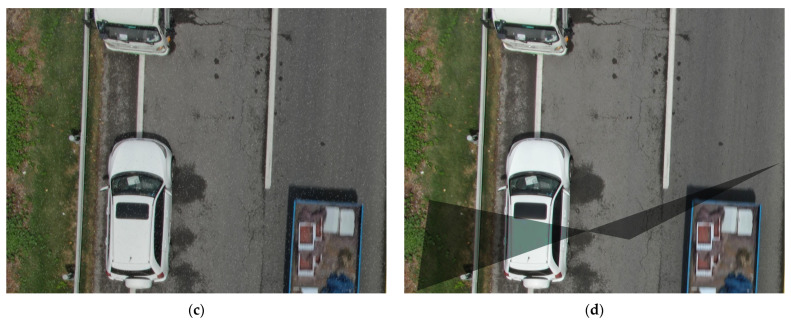
Example images under various conditions: (**a**) original image; (**b**) nighttime image; (**c**) rainy condition image; (**d**) occlusion scenario image.

To thoroughly evaluate the robustness of the improved model under realistic and complex scenarios, we applied data augmentation to the test set using the Albumentations library. This approach simulates various real-world conditions, such as varying illumination, weather conditions, and occlusions. Examples of augmented images are shown in Figure 8.

Under the aforementioned complex conditions, we evaluated the models using two metrics: mAP50 and Performance Drop. Here, Performance Drop represents the percentage decrease in mAP50 when the model is tested under complex scenarios compared to standard conditions. As shown in Table 5, the Performance Drop of RT-DETR under varying illumination, weather, and occlusion conditions is 2.52%, 11.08%, and 10.2%, respectively, while Pavement-DETR achieves notably lower values of 0.57%, 7.46%, and 7.12%. This clearly indicates that Pavement-DETR exhibits superior robustness in complex, real-world scenarios.

## 5. Conclusions

Pavement Management Systems play a vital role in maintaining safe and efficient road infrastructure. These systems confront various challenges, such as detecting small targets, handling overlapping targets, and dealing with complex backgrounds, which collectively impact their accuracy and effectiveness. In response to these challenges, this paper proposes a novel road defect detector, Pavement-DETR, based on RT-DETR, focusing on improving accuracy. First, we introduce the CSS attention mechanism into multi-scale features, significantly enhancing the model’s feature extraction capability and effectively reducing false positives and false negatives. Additionally, we incorporate the Conv3XC module, which strengthens the model’s ability in feature fusion, helping the model better distinguish the foreground and background of the road. Finally, we propose an improved loss function that effectively enhances the model’s ability to capture small targets and focuses on almost right areas. The integration of these three key innovations has formed Pavement-DETR. Extensive testing on the UAV-PDD2023 dataset shows that Pavement-DETR, compared to the baseline RT-DETR model, significantly improves the accuracy of road defect detection. It achieves a mean average precision increase of 7.7% at an IoU of 0.5 and 8.9% across IoU thresholds from 0.5 to 0.95. Moreover, Pavement-DETR maintains a good balance between computational cost and detection accuracy, making it a practical and efficient solution for road maintenance work.

Firstly, we integrate a CSS attention mechanism into our multi-scale feature processing, which markedly improves the model’s capability to extract relevant features while significantly reducing both false positives and false negatives. Furthermore, we have added the Conv3XC module to enhance feature fusion, aiding the model in more effectively differentiating between the road’s foreground and background. In addition, an upgraded loss function has been developed to better detect small targets and to refine focus on medium quality anchor boxes. These three major advancements constitute the essence of Pavement-DETR. Extensive evaluations on the UAV-PDD2023 dataset have demonstrated that Pavement-DETR substantially enhances road defect detection accuracy compared to the baseline RT-DETR model. Specifically, it achieves a mean average precision increase of 7.7% at an IoU threshold of 0.5, and 8.9% across various IoU thresholds from 0.5 to 0.95. Pavement-DETR also maintains an optimal balance between computational cost and detection performance, establishing it as a practical and effective tool for road maintenance operations.

Despite the significant improvements in accuracy achieved by Pavement-DETR in road defect detection, the model still has some limitations. For example, the detection accuracy may decline in foggy or low-light conditions. In the future, we will continue to focus on the field of road defect detection, aiming to enhance the model’s robustness in various complex environments, thereby improving its detection performance under different weather conditions. Additionally, we will further explore lightweight strategies for the model to achieve faster inference speeds and lower computational resource consumption, providing more valuable data for detecting road defects, which assists in developing practical road maintenance strategies.

## Figures and Tables

**Table 1:** Experimental environment.

Configuration	Parameter
Operating System	Ubuntu 20.04
Memory	256 GB
CPU	Intel Xeon Silver 4316
GPU	NVIDIA A30
GPU Memory	24 G
Programming Language	Python 3.10.9
CUDA Version	CUDA 12.1
Deep Learning Framework	Pytorch 2.1.2

**Table 2:** Training parameters.

Parameter	Value
Epoch	900
Batch Size	16
Workers	8
Input Size	640 × 640
Optimizer	AdamW
Initial Learning Rate	0.0001
Momentum	0.9
Weight Decay Coefficient	0.0001

**Table 3:** Performance comparison of different networks, where bold indicates better performance.

Algorithm	P/%	R/%	mAP@0.5%	mAP@0.5:0.95%	FLOPs/G
RT-DETR-R18	0.861	0.769	0.794	0.509	57.0
YOLOv8s	0.834	0.713	0.793	0.482	28.4
YOLOv8m	0.848	0.691	0.791	0.523	78.7
YOLOv9s	0.802	0.674	0.74	0.459	26.7
YOLOv9m	0.833	0.767	0.811	0.564	76.5
YOLOv10s	0.826	0.772	0.825	0.545	**24.5**
YOLOv10m	0.861	0.622	0.73	0.489	63.4
Deformable-DETR	0.848	0.737	0.776	0.43	165
Dab-DETR	0.869	0.711	0.767	0.407	86.9
Conditional-DETR	0.881	0.695	0.766	0.405	85.6
Dino-DETR	0.864	0.752	0.797	0.535	235
Ours	**0.893**	**0.838**	**0.871**	**0.598**	67.5

**Table 4:** Ablation experimental results, where √ indicates the use of the corresponding improvement module.

CSS	Conv3XC	Loss	AP%						mAP @0.5%	mAP @0.5:0.95%	F1 Score/%	FLOPs/G
			D1	D2	D3	D4	D5	D6				
			0.825	0.837	0.79	0.816	0.614	0.886	0.794	0.509	0.80	57.0
√			0.847	0.866	0.799	0.827	0.678	0.862	0.813	0.548	0.81	67.5
√	√		0.871	0.859	0.844	0.838	0.737	0.962	0.852	0.584	0.86	67.5
√	√	√	0.888	0.905	0.871	0.884	0.704	0.976	0.871	0.598	0.87	67.5

**Table 5:** Detection performance and robustness of models in different scenarios, where bold indicates better performance.

Model	Normal mAP@0.5%	Brightness mAP@0.5%	Performance Drop (%)	Rain mAP@0.5%	Performance Drop (%)	Shadow mAP@0.5%	Performance Drop (%)
RT-DETR	0.794	0.774	2.52	0.706	11.08	0.713	10.2
Ours	0.871	0.866	**0.57**	0.806	**7.46**	0.809	**7.12**

## Data Availability

The data presented in this study are available on request from the corresponding author.
